# Relation of Dietary Fatty Acids and Vitamin D to the Prevalence of Meibomian Gland Dysfunction in Japanese Adults: The Hirado–Takushima Study

**DOI:** 10.3390/jcm10020350

**Published:** 2021-01-18

**Authors:** Shima Fukuoka, Reiko Arita, Takanori Mizoguchi, Motoko Kawashima, Shizuka Koh, Rika Shirakawa, Takashi Suzuki, Satoshi Sasaki, Naoyuki Morishige

**Affiliations:** 1 Lid and Meibomian Gland Working Group (LIME), 626-11 Minami-Nakano, Minumaku, Saitama 337-0042, Japan; fshima3271@gmail.com (S.F.); t-mizo@siren.ocn.ne.jp (T.M.); motoko326@gmail.com (M.K.); cizciz@gmail.com (S.K.); rikadream@hotmail.com (R.S.); takashisuzuki58@gmail.com (T.S.); morishig@corneajp.com (N.M.); 2 Omiya Hamada Eye Clinic, 1-169-1 Sakuragicho, Omiyaku, Saitama 330-0854, Japan; 3 Department of Ophthalmology, The University of Tokyo, 7-3-1 Hongo, Bunkyoku, Tokyo 113-8655, Japan; 4 Department of Ophthalmology, Itoh Clinic, 626-11 Minami-Nakano, Minumaku, Saitama 337-0042, Japan; 5 Mizoguchi Eye Clinic, 6-13 Tawaramachi, Sasebo, Nagasaki 857-0016, Japan; 6 Department of Ophthalmology, Keio University, 35 Shinanomachi, Shinjukuku, Tokyo 160-8582, Japan; 7 Department of Ophthalmology, Osaka University, 2-15 Yamadaoka, Suita 565-0871, Japan; 8 Department of Ophthalmology, Toho University Omori Medical Center, 6-11-1 Omorinishi, Otaku, Tokyo 143-8541, Japan; 9 Department of Social and Preventive Epidemiology, School of Public Health, The University of Tokyo, 7-3-1 Hongo, Bunkyoku, Tokyo 113-0033, Japan; stssasak@m.u-tokyo.ac.jp; 10 Division of Cornea and Ocular Surface, Ohshima Eye Hospital 11-8 Kamigofukumachi, Hakataku, Fukuoka 812-0036, Japan

**Keywords:** fatty acids, vitamin D, meibomian gland dysfunction, brief-type self-administered diet history questionnaire, Japanese

## Abstract

Intervention studies have shown that n-3 polyunsaturated fatty acid (PUFA) supplementation is effective for the treatment of meibomian gland dysfunction (MGD). Ointment containing an analog of vitamin D has also been found to improve symptoms and signs of MGD. We have now evaluated the relation of MGD prevalence to dietary intake of fatty acids (FAs) and vitamin D among a Japanese population. Subjects comprised 300 adults aged 20 to 92 years residing on Takushima Island. MGD was diagnosed on the basis of subjective symptoms, lid margin abnormalities, and meibomian gland obstruction. Dietary FA and vitamin D intake was estimated with a brief-type self-administered diet history questionnaire. MGD prevalence was 35.3%. Multivariate adjusted odds ratios (95% confidence intervals) between extreme quintiles of intake for MGD prevalence were 0.40 (0.16–0.97) for total fat, 0.40 (0.17–0.97) for saturated FAs, 0.40 (0.17–0.97) for oleic acid, 0.52 (0.23–1.18) for n-3 PUFAs, 0.63 (0.27–1.49) for n-6 PUFAs, 1.32 (0.59–2.95) for the n-6/n-3 PUFA ratio, and 0.38 (0.17–0.87) for vitamin D. Total fat, saturated FA, oleic acid, and vitamin D intake may thus be negatively associated with MGD prevalence in the Japanese.

## 1. Introduction

The ocular surface is covered and protected by the tear film comprised of lipid, aqueous, and mucin layers. Meibomian glands are sebaceous glands in the eyelids and secrete meibum, which is composed of >600 types of lipid [1], most of which are unsaturated wax esters and derived from oleic acid [2]. Meibum forms the lipid layer of the tear film, which plays an important role in the stabilization of the tear film [3,4] and prevents excessive evaporation of tear fluid [5]. Meibomian gland dysfunction (MGD) is defined by the International Workshop on Meibomian Gland Dysfunction as “a chronic, diffuse abnormality of the meibomian glands that is commonly characterized by terminal duct obstruction or qualitative or quantitative changes in glandular secretion” [6]. MGD is thus associated with tear film instability as a result of deficiency of the lipid layer. MGD is also a major cause of dry eye disease—in particular, of evaporative dry eye [6,7,8]. Population-based studies have revealed prevalence rates for MGD ranging from 38% to 68% among individuals over the age of 40 years [9]. We previously found that the prevalence of symptomatic MGD was 32.9% among Japanese individuals aged 6 years or older and 44.5% among those aged 40 years or older in a population-based study (Hirado-Takushima study) [10].

Therapies for MGD aim to improve the quality and quantity of meibum, to stabilize the tear film, to reduce inflammation, and to ameliorate subjective symptoms. Dietary supplementation with n-3 polyunsaturated fatty acids (PUFAs) is recommended both by the International Workshop on Meibomian Gland Dysfunction [11] and by the International Dry Eye Workshop [12]. Given that the human body is not able to synthesize n-3 or n-6 PUFAs, these essential fatty acids must be obtained from the diet. Several randomized, double-masked trials found that n-3 PUFA supplementation improved subjective symptoms [13,14,15], tear film stability [13,14,15], lid margin telangiectasia [13,14], as well as meibum quality [13,15] and expressibility [14,15] in individuals with MGD. A cross-sectional study also reported that dietary intake of n-3 PUFAs was negatively related and that the n-6/n-3 PUFA ratio was positively related to the prevalence of dry eye in women [16]. However, another cross-sectional study found that neither dietary intake of n-3 or n-6 PUFAs nor the n-6/n-3 PUFA ratio was associated with dry eye in postmenopausal women [17]. This latter study did detect a negative relation between dietary intake of n-3 and n-6 PUFAs and the prevalence of MGD [17].

Topical application of an analog of the active form of vitamin D3 was shown to ameliorate ocular symptoms and objective signs of MGD compared with baseline [18]. Oral vitamin D supplementation was also found to improve meibomian gland expressibility in MGD patients with vitamin D deficiency [19]. As far as we are aware, however, no observational study has previously examined the relationship between dietary intake of vitamin D and MGD.

Given the paucity of evidence regarding the relation between diet and MGD, we have now performed a cross-sectional study to investigate the relation of dietary consumption of specific types of fatty acids and vitamin D to the prevalence of MGD among adult Japanese in the Hirado-Takushima study.

## 2. Experimental Section

### 2.1. Study Design and Subjects

The present study is based on the Hirado-Takushima study, a population-based cross-sectional study that investigated the prevalence and risk factors of MGD and dry eye in Japan [10]. The subjects of this cross-sectional study were adult residents of Takushima Island, Hirado, Nagasaki, Japan, and the study adhered to the tenets of the Declaration of Helsinki and was approved by the Institutional Review Board of Itoh Clinic and registered in the University Hospital Medical Information Network database (UMIN000028310). Written informed consent was obtained from all participants before inclusion in the study. The dietary and lifestyle assessments of the study subjects were based on questionnaires conducted in August 2017. The ocular assessments were performed in November 2017. A total of 338 adult residents (119 men, 219 women) was recruited to the study. A set of three self-administered questionnaires was completed by the participants at home. Responses to the questionnaires were checked once by a physician who did not participate in the ocular examinations. If any missing or erroneous responses to questions considered essential for the analysis were detected, the subject was asked to complete or correct those responses. Exclusion criteria for the present study included pregnancy (*n* = 0) or nursing (*n* = 8) for women, an extremely low (<500 kcal/day, *n* = 2) or high (>4000 kcal/day, *n* = 0) reported energy intake, missing data for ophthalmologic measurements included in multivariate analysis (*n* = 27), and missing answers to the diet history questionnaire (*n* = 1). The final analysis was thus performed for 300 residents (109 men, 191 women) aged 20 to 92 years.

### 2.2. Dietary Assessment

Dietary habits during the preceding month were assessed with a brief-type self-administered diet history questionnaire (BDHQ) that was designed to assess the habitual dietary intake of Japanese adults and had been previously validated [20,21]. Details of the structure of the questionnaire, its method for calculation of dietary intake, and its validity for commonly studied food and nutrient intake have been described previously [20,21]. Participants were asked about the frequency of intake for food and nonalcoholic beverage items, daily intake of rice and miso soup, frequency and amount of alcoholic beverage consumption, and usual cooking methods. The BDHQ allows estimation of the daily intake of 58 food items, energy, and specific nutrients, including fats and vitamins, on the basis of the Standard Tables of Food Composition in Japan [22]. It is a four-page fixed-portion questionnaire. To facilitate reading and completion of the BDHQ, the present study adopted a large-print version that increased the printed size to 10 pages but which contained no other changes to the structure or content for participants aged >55 years. Fish and shellfish, meat, eggs, and dairy products were included in animal food. Cereals, pulses, potatoes, confectionaries, fruits, vegetables, and seasonings and spices were included in plant food. Dietary supplements were not included in the calculation of nutrient or fatty acid intake because of the lack of a reliable composition table for dietary supplements in Japan. Supplement use was queried in the BDHQ and treated as a confounding factor. The percentage contribution of each food group to total fat or vitamin D intake was calculated by dividing daily fat or vitamin D intake from each food group by total daily consumption of fat or vitamin D.

### 2.3. Assessment of Subjective Symptoms and Other Variables

Subjective ocular symptoms related to dry eye were assessed with the Dry Eye-Related Quality-of-Life Score (DEQS) [23]. The subjects reported their birth date, body weight, and height in the BDHQ. Body mass index (BMI) was calculated by dividing self-reported body weight (kg) by the square of self-reported height (m^^2^^). A questionnaire on lifestyle also elicited information on occupation, history of ocular surgery, eyedrop use, contact lens wear, history of chronic systemic disease, history of dyslipidemia, taking of lipid-lowering agents, smoking and alcohol habits, and taking of supplements. Details were described previously [10].

### 2.4. Ocular Examinations

The ocular surface of both eyes was assessed according to standardized protocols by a team of seven ophthalmologists with expertise in dry eye and MGD (members of the Lid and Meibomian Gland Working Group) [10]. Participants were diagnosed with MGD [24] on the basis of (1) the presence of any chronic ocular symptom [23]; (2) the detection of more than one lid margin abnormality among vascularity, displacement of the mucocutaneous junction, and irregularity; and (3) obstruction of meibomian glands as revealed by the detection of plugging and reduced meibum expression in response to moderate digital pressure [25] in at least one eye. The ophthalmologists performing the examinations remained masked to the responses to the BDHQ throughout the assessment.

### 2.5. Statistical Analysis

All dietary variables were adjusted for energy by the residual method with the use of a linear regression model. The data were found to be non-normally distributed with the Shapiro–Wilk test (*p* < 0.05), and nonparametric testing was thus performed. The characteristics of the subjects with or without MGD were compared with the Mann–Whitney *U* test for continuous variables or Fisher’s exact test for categorical variables.

The intake of specific nutrients adjusted by the residual method was categorized at quintile points on the basis of the distribution for all study subjects. The odds ratio (OR) and its 95% confidence interval (CI) for fat, fatty acids, the n-6/n-3 PUFA ratio, and vitamin D with regard to MGD were calculated by different logistic regression models after adjustment for potential confounding factors: age, sex, BMI, energy intake, occupation, history of ocular surgery, eyedrop use, contact lens wear, history of chronic systemic disease, history of dyslipidemia, taking of lipid-lowering agents, current smoking, alcohol habit, and taking of supplements, with the lowest quintile as the reference. The initial logistic regression model was a crude model (that is, an unadjusted model), to which covariates were added with a forward selection method. In a second multivariate model (Model 1), age (years, continuous) and sex (male or female) were used as potential confounding factors. The final multivariate model (Model 2) included age (years, continuous), sex (male or female), BMI (kg/m^2^, continuous), history of chronic systemic disease (yes or no), and alcohol consumption (yes or no) as potential confounding factors. Other variables—energy intake (kcal/day, continuous), occupation (fisherman, farmer, local government official, or other), history of ocular surgery (yes or no), eyedrop use (yes or no), contact lens wear (yes or no), history of dyslipidemia (yes or no), taking of lipid-lowering agents (yes or no), current smoking (yes or no), and taking of supplements (yes or no)—were not included in Models 1 and 2 because they had no influence on the relationship between dietary variables and MGD (*p* > 0.10).

Data are presented as means ± standard deviations. Statistical analysis was performed with JMP Pro version 14 software (SAS, Cary, NC, USA), all statistical tests were two sided, and a *p* value of <0.05 was considered statistically significant.

## 3. Results

### 3.1. Subsection

Table 1 shows the distribution of selected variables among all subjects as well as in the non-MGD and MGD groups. A total of 106 subjects (35.3%) was diagnosed with MGD. Mean age of the study population was 61.7 ± 15.6 years, and mean BMI was 23.9 ± 3.5 kg/m^^2^^. Compared with the non-MGD group, the MGD group was significantly older (*p* < 0.001) and included a larger proportion of men (*p* = 0.006). BMI did not differ significantly between the two groups (*p* = 0.99). A higher proportion of subjects with MGD had a history of chronic systemic disease (*p* < 0.001). No significant differences between the two groups were apparent for history of dyslipidemia (*p* = 0.39), taking of lipid-lowering agents (*p* = 1.0), or taking of dietary supplements (*p* = 0.89).

### 3.2. Daily Intake of Fatty Acids and Vitamin D in Subjects with or Without MGD

Table 2 shows the daily intake of specific dietary components in all subjects as well as in those of the non-MGD or MGD groups, with *p* values for comparisons between the latter two groups being determined with the Mann–Whitney *U* test. Mean intake of total fat, animal fat, or plant fat for all subjects was 50.7 ± 11.7, 23.3 ± 8.3, and 27.4 ± 7.2 g/day, respectively. The mean intake of fatty acids was 13.7 ± 4.0 g/day for saturated fatty acids (SFAs), 18.1 ± 4.5 g/day for monounsaturated fatty acids (MUFAs), 12.3 ± 2.9 g/day for PUFAs, 2.6 ± 1.0 g/day for n-3 PUFAs, and 9.7 ± 2.4 g/day for n-6 PUFAs. Mean intake of the MUFA oleic acid was 16.4 ± 4.1 g/day, whereas that of the n-3 PUFAs eicosapentaenoic acid (EPA) and docosahexaenoic acid (DHA) was 0.33 ± 0.27 and 0.55 ± 0.40 g/day, respectively. Mean intake of vitamin D was 15.4 ± 11.9 μg/day. Intake of total fat and animal fat was significantly lower in the MGD group than in the non-MGD group (*p* = 0.007 and 0.002, respectively). There was no significant difference in plant fat intake between the two groups (*p* = 0.60). Intake of SFAs, MUFAs, PUFAs, and n-3 PUFAs was also significantly lower in the MGD group than in the non-MGD group (*p* = 0.015, 0.005, 0.039, and 0.017, respectively), as was intake of oleic acid and EPA (*p* = 0.007 and 0.044, respectively). There was no significant difference in n-6 PUFA intake or in the n-6/n-3 PUFA ratio between the two groups (*p* = 0.10 and 0.40, respectively). Vitamin D intake in the MGD group was significantly lower than that in the non-MGD group (*p* = 0.039). The main contributors to total fat intake in the study population were cooking oil (20%), meat (15%), fish and shellfish (12%), and dairy products (11%), whereas those to vitamin D intake were fish and shellfish (92%) and eggs (4%) (Table 3).

### 3.3. Multivariate Adjusted ORs for Fatty Acid and Vitamin D Intake with Regard to MGD

**Figure 1:**
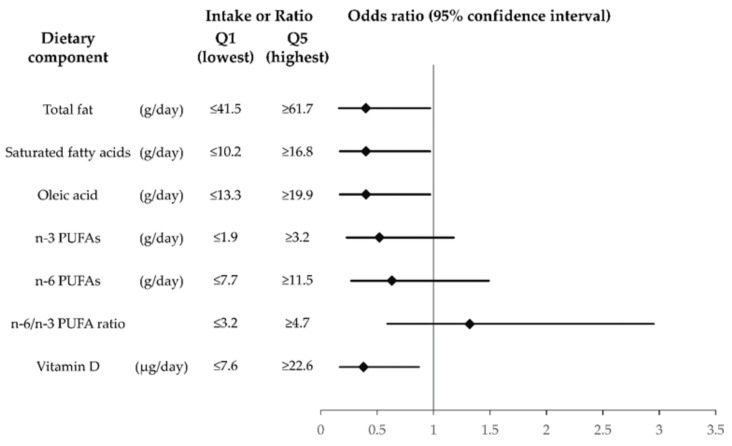
Multivariate adjusted odds ratios and 95% confidence intervals for the prevalence of meibomian gland dysfunction according to extreme quintiles (Q1 and Q5) of intake of specific fats and vitamin D among adult subjects in the Hirado–Takushima study. Nutrient intake was adjusted for energy by the residual method. The results are for Model 2, with adjustment for age (years, continuous), sex (male or female), body mass index (kg/m2, continuous), history of chronic systemic disease (yes or no), and alcohol drinking (yes or no). PUFA, polyunsaturated fatty acid.

The ORs and 95% CIs for the prevalence of MGD according to dietary intake of specific types of fatty acids and vitamin D are shown in Table 4 and Figure 1. Intake of SFAs was inversely associated with the prevalence of MGD after adjustment for sex and age (*p* for trend = 0.026, Model 1), and this association was maintained after further multivariate adjustment (*p* for trend = 0.020, Model 2). The multivariate adjusted ORs and 95% CIs for MGD prevalence in the first, second, third, fourth, and fifth quintiles of SFA intake were 1.00 (reference), 0.34 (0.15– 0.78), 0.44 (0.19–1.02), 0.94 (0.42–2.13), and 0.40 (0.17–0.97), respectively, in Model 2. Intake of n-3 PUFAs was also inversely associated with MGD after adjustment for sex and age (*p* for trend = 0.049, Model 1), but this association was not maintained after further multivariate adjustment (*p* for trend = 0.077, Model 2) and none of the multivariate adjusted ORs for the second to fifth quintiles differed significantly relative to the first quintile. The multivariate adjusted ORs and 95% CIs for the fifth quintile compared with the first quintile in Model 2 were 0.40 (0.16–0.97) for total fat, 0.40 (0.17–0.97) for oleic acid, and 0.38 (0.17–0.87) for vitamin D. For EPA, the multivariate adjusted OR (and 95% CI) for the fourth quintile compared with the first quintile in Model 2 was 0.41 (0.17–0.97), but that in the fifth quintile compared with the first quintile was 0.68 (0.31–1.50). Whereas intake of animal fat, MUFAs, or PUFAs was significantly lower in the MGD group than in the non-MGD group by the Mann–Whitney *U* test (Table 2), none of these dietary components was significantly related to the prevalence of MGD in either Model 1 or 2 of the multivariate analysis (Table 4). There was no significant difference in intake of plant fat, α-linolenic acid, DHA, n-6 PUFAs, linoleic acid, arachidonic acid, or cholesterol or in the n-6/n-3 PUFA ratio between the MGD and non-MGD groups by the Mann–Whitney *U* test (Table 2), and there was no significant relationship between these parameters and the prevalence of MGD in Model 1 or 2 of the multivariate analysis (Table 4).

## 4. Discussion

Our cross-sectional study investigated the relation of dietary intake of fatty acids and vitamin D to the prevalence of MGD. The results revealed that SFA intake was inversely associated with MGD prevalence (*p* for trend = 0.020, Model 2) among adult residents of Takushima Island, and that the highest quintiles for dietary intake of total fat, SFAs, oleic acid, and vitamin D were significantly associated with a lower prevalence of MGD compared with the first quintiles (Model 2). There was no significant association between either the intake of n-3 PUFAs, n-6 PUFAs, or cholesterol or the n-6/n-3 PUFA ratio and the prevalence of MGD by multivariate logistic regression analysis.

Diet and oral supplementation are different things. In our study, multivariate logistic regression analysis revealed that there was no significant association between intake of n-3 PUFAs or n-6 PUFAs or the n-6/n-3 PUFA ratio and the prevalence of MGD. A previous cross-sectional study found that the prevalence of MGD was 21.9% in 319 postmenopausal women and that high n-3 PUFA intake (OR and 95% CI, 0.22 and 0.06–0.78) and moderate n-6 PUFA intake (0.37 and 0.15–0.91) were significantly associated with a lower prevalence of MGD [17]. In this previous study, however, only postmenopausal women 50 years of age and older were recruited and the diagnosis of MGD was based only on reduced meibum expressibility with digital pressure for the 10 central meibomian glands of the lower eyelids [26]. In our study, we recruited both men and women at least 20 years of age and diagnosed MGD on the basis of subjective symptoms, lid margin abnormalities, and obstruction of meibomian glands [24]. In the MGD group of the previous study [17], the mean daily intake of n-3 PUFAs (1.87 g/day) was lower, that of n-6 PUFAs (15.24 g/day) was higher, and the mean n-6/n-3 PUFA ratio (8.35) was higher compared with the corresponding values for the MGD group of our study (2.5 g/day, 9.3 g/day, and 4.0, respectively). Another previous cross-sectional study of the Mediterranean diet found that the daily intake of n-3 PUFAs was not significantly associated with tear film breakup time as measured with fluorescein (OR and 95% CI, 0.87 and 0.38–2.01), with meibum quality (1.06 and 0.48–2.39), or with plugging of the inferior eyelid margin (0.91 and 0.37–2.20) in 247 men aged 55 to 95 years [27]. Although meibomian gland parameters were assessed and dry eye was diagnosed in this previous study, the diagnosis of MGD was not made [27]. The typical Western diet is heavy on red and processed meats, poultry, and full-fat dairy products, and it tends to be low in n-3 PUFAs, to be high in n-6 PUFAs, and to have a high n-6/n-3 PUFA ratio of between 15 and 25 [28,29]. In contrast, the Mediterranean-style diet is characterized by a high intake of non-refined cereals, fruits, vegetables, legumes, olive oil, fish, and potatoes and a low intake of SFAs [30,31], with a low n-6/n-3 PUFA ratio of ~4 [13]. The traditional Japanese diet is characterized by a high intake of fish and plant foods and a low intake of refined carbohydrates and meat [32]. The participants of our study had a relatively high n-3 PUFA intake, low n-6 PUFA intake, and low n-6/n-3 PUFA ratio. An inverse relationship between n-3 PUFA intake and the prevalence of MGD, and the lack of an association between the n-6/n-3 PUFA ratio and MGD prevalence, might therefore be apparent only when consumption of n-3 PUFAs is low.

There have been only three observational studies that have assessed the relation of dietary intake of fatty acids to the prevalence of dry eye. A cross-sectional study with a large cohort of female health professionals (the Women’s Health Study) showed an association between lower dietary intake of n-3 PUFAs and a higher n-6/n-3 PUFA ratio and a higher prevalence of dry eye [16]. There was no association between n-6 PUFA intake and dry eye prevalence. Dry eye in this study was defined on the basis of asking participants whether they had been clinically diagnosed with dry eye syndrome [16], and MGD was not assessed. Two other cross-sectional studies found that dietary intake of n-3 PUFAs was not associated with the prevalence of dry eye disease [17,27]. An interventional study showed that consumption of a Mediterranean diet for 6 months was associated with amelioration of subjective symptoms of dry eye, an increase in both the fluorescein breakup time of the tear film (FBUT) and Schirmer’s test value, and reduced fluorescein staining of the ocular surface [30]. There is a consensus that dry eye and MGD are similar and overlapping diseases. We previously showed that the ocular symptoms of MGD and dry eye are similar, but that the pathogenesis and risk factors of the two conditions differ [10]. The results of research on dry eye thus need to be considered separately from those of studies on MGD. The Hirado–Takushima study was designed to focus on dry eye and MGD, and we therefore believe that the current study is equipped to specifically reveal the relation between diet and MGD prevalence.

Several randomized, controlled studies have investigated the efficacy of n-3 PUFA supplements for MGD [13,14,15,33]. A prospective, randomized, double-masked trial found that n-3 PUFA supplementation with EPA at 1050 mg/day and DHA at 127.5 mg/day together with the practice of lid hygiene and administration of preservative-free artificial tears for 3 months improved subjective symptoms, FBUT, lid margin inflammation, and meibomian gland expression compared with baseline [14]. The placebo (sunflower oil) together with lid hygiene and preservative-free artificial tears ameliorated subjective symptoms but did not change other objective parameters [14]. A prospective, randomized, placebo-controlled, masked trial showed that dietary supplementation with EPA at 720 mg/day and DHA at 480 mg/day for 12 weeks improved contrast sensitivity under photopic and mesopic testing conditions, subjective symptoms, FBUT, the fluorescein staining score, meibum expressibility, and meibum quality in patients with moderate MGD compared with the placebo group treated with vitamin E at 400 mg/day [15]. Another such trial showed that dietary supplementation with EPA at 1680 mg/day and DHA at 560 mg/day for 12 weeks improved subjective symptoms, tear osmolarity, and FBUT compared with the placebo group treated with linoleic acid (safflower oil) at 3136 mg/day [34]. Yet another trial showed that dietary supplementation with n-3 PUFAs at 3 g/day for 12 months improved subjective symptoms, FBUT, lid margin telangiectasia, meibum quality, meibomian gland blockage, and the number of visible ducts of meibomian glands compared with baseline in patients with blepharitis and obstructive MGD [13]. On the other hand, the placebo (olive oil) improved FBUT, meibum quality, meibomian gland stenosis, and the number of visible ducts of meibomian glands. The changes in FBUT and meibum quality did not differ significantly between the n-3 PUFA and placebo groups [13]. A prospective, randomized, multicenter clinical trial (Dry Eye Assessment and Management, or DREAM, trial) recently failed to detect a superior improvement in subjective symptoms and objective parameters of dry eye in individuals receiving n-3 PUFAs at 3 g/day (EPA at 2 g/day and DHA at 1 g/day) compared with those receiving placebo (olive oil at 5 g/day) [35]. There were no significant differences in the corneal and conjunctival staining score, FBUT, or Schirmer’s test value between the n-3 PUFA supplement group and the placebo group [35]. Parameters related to meibomian glands were not assessed in this previous study. In our study, the multivariate adjusted OR and 95% CI for MGD prevalence and the highest quintile of oleic acid intake compared with the lowest quintile were 0.40 and 0.17 to 0.97 in Model 2. This result suggests the possibility that oleic acid intake may protect against the development of MGD. Gas chromatography revealed that the major components of olive oil were oleic acid (C18:1cis, n-9) at 66.4%, palmitic acid (C16:0) at 16.5%, and linoleic acid (C18:2cis, n-6) at 16.4%, whereas those of sunflower oil were linoleic acid at 62.2% and oleic acid at 28.0% [36]. Oleic acid has been shown to be a major constituent of meibum [2,37,38] and to protect against oxidative stress [39]. The use of olive oil containing oleic acid as a placebo might thus have influenced the results of previous studies of the relation between n-3 PUFA supplementation and MGD or dry eye [13,35]. The average dietary intake of n-3 PUFAs was 2.6 ± 1.0 g/day for all subjects of our study, with values of 1.5 ± 0.4 and 4.0 ± 1.0 g/day for those in the lowest and highest quintiles, respectively. The participants of our study thus had an n-3 PUFA intake similar to that for patients of previous interventional studies with n-3 PUFA supplements. It is possible that we did not detect a protective effect of n-3 PUFAs on MGD in our study as a result of the relatively high intake even in the lowest intake group.

Both n-3 PUFAs and n-6 PUFAs are essential fatty acids and have been shown to play an important role in regulation of inflammatory and immune responses. The metabolic pathways for n-3 and n-6 PUFAs share enzymes with the inflammation-related metabolism of arachidonic acid. Whereas n-3 PUFAs are thought to be anti-inflammatory, n-6 PUFAs are thought to inhibit this function of n-3 PUFAs in a competitive manner [12,13,29]. The role of inflammation in MGD is not clearly understood [40], but n-3 PUFA intake may reduce the inflammatory state of the eyelid margin.

The balanced composition of meibum is important for maintenance of the stability of the tear film [38,41]. The oleic acid content of meibum was shown to be higher in patients with meibomian seborrhea than in those with meibomianitis or in healthy individuals [41]. Fatty acids in meibum of MGD patients were found to include a higher proportion of branched-chain fatty acids and a lower proportion of SFAs, especially of palmitic (C16) and stearic (C18) acids, compared with those in healthy meibum [38]. Supplementation with n-3 PUFAs increased n-3 fatty acid levels and reduced the n-6/n-3 fatty acid ratio in both plasma [13] and red blood cells [13,35] compared with placebo. A randomized, controlled-feeding, double-blind, crossover study reported that a diet containing oil with a high level of oleic acid lowered circulating concentrations of total cholesterol, low-density-lipoprotein cholesterol, apolipoprotein B, and non-high-density-lipoprotein cholesterol compared with a diet containing a Western-type control oil with a low level of oleic acid [42]. Previous studies have revealed an association between dyslipidemia and MGD [10,43,44,45,46,47]. In the current study, no difference in the history of dyslipidemia (*p* = 0.39) or in the taking of lipid-lowering agents (*p* = 1.0) was apparent between the non-MGD and MGD groups. In our previous report of the Hirado–Takushima study, however, we found that the use of lipid-lowering agents was significantly and independently associated with MGD (OR and 95% CI of 3.22 and 1.05–9.87) by multivariate logistic regression analysis [10]. Exposure to n-3 and n-6 PUFAs for up to 7 days in vitro was found to increase the quantity of intracellular small secretory lipid vesicles and the cellular content of triglycerides in human meibomian gland epithelial cells [48]. Dietary intake of n-3 PUFAs and other unsaturated fatty acids was also found to be associated with a significant change in the lipid profile of meibum in 18 women with Sjögren’s syndrome [49]. Dietary fatty acid intake and its balance may thus affect not only the composition of lipids in blood but also that of meibum.

We found that the multivariate adjusted OR (and 95% CI) for the prevalence of MGD and the fifth quintiles of total fat or SFAs in Model 2 was 0.40 (0.16–0.97) and 0.40 (0.17–0.97), respectively, suggesting that a high intake of total fat and SFAs might protect against MGD. As far as we are aware, the relation between total fat or SFA intake and MGD has not previously been examined. Dietary guidelines recommend that SFA intake be limited to <10% of energy intake or be as low as possible in order to reduce ischemic heart disease and stroke [50,51]. Recent meta-analyses, however, found that SFAs were not associated with cardiovascular disease [52,53], coronary heart disease [52], ischemic stroke [52], type 2 diabetes [52], or breast cancer [54]. Another meta-analysis showed that odd-chain SFAs reduced the risk of type 2 diabetes, whereas even- and very long-chain SFAs increased it [55]. Total fat was also not associated with cardiovascular disease [53] or breast cancer [54] in previous meta-analyses. The human body is made up of nutrients from the diet. The quantity and quality of lipids secreted from meibomian glands is altered in MGD [7]. Although the role of fatty acid intake and its balance in MGD is not clearly understood, excessive, insufficient, or unbalanced intake of dietary fatty acids may induce MGD. Further research is needed to clarify the role of dietary fatty acid intake in MGD.

Serum levels of 25-hydroxyvitamin D have been associated with dry eye [27,56,57]. There has been no previous study of the relation between dietary intake or serum levels of vitamin D and MGD. In our present study, the highest quintile of vitamin D intake was associated with a low prevalence of MGD. A previous case study reported that oral vitamin D supplementation ameliorated persistent symptoms in a 40-year-old patient with ocular pain, bilateral MGD, evaporative dry eye, and vitamin D deficiency [58]. A single-arm clinical study of 40 patients with vitamin D deficiency found that oral vitamin D supplementation for 8 weeks improved meibomian gland expressibility, eyelid margin condition, Schirmer’s test value, FBUT, and subjective symptoms compared with baseline [19]. Eight-week topical application of an analog of the active form of vitamin D3 was also found to be associated with improved ocular symptoms, plugging and vascularity of lid margins, FBUT, corneal fluorescein staining, meibum grade, and meibomian gland area in patients with obstructive MGD [18]. Recent studies have provided a new insight into the physiological role of vitamin D in extra skeletal tissues [59]. Hyperkeratinization is thought to be a major cause of obstructive MGD [40,60], and the active form of vitamin D3 was found to inhibit the proliferation of keratinocytes and to promote their differentiation [61,62,63,64]. Vitamin D and the vitamin D receptor are also implicated in the control of inflammation and immunity [59,65], as well as in that of lipid metabolism [66]. Vitamin D deficiency has been found to be related to dyslipidemia [67,68]. Meibum is composed of many types of lipid. Dietary vitamin D intake might thus be expected to be associated with MGD. Further studies focusing on the relation between vitamin D and MGD may help to elucidate the pathogenesis of MGD and develop new treatment strategies for this condition.

The present study has several limitations. First, many of the male subjects work as fishermen but fish away from the island. Our results thus cannot be readily extended to the general Japanese population. Epidemiological studies in urban areas may yield different results. We would like to do another population-based cross-sectional study in an urban area of Japan and compare the results with the present study in the future. Second, the BDHQ is a self-reported diet history questionnaire. We excluded subjects reporting low or high energy intake, and we used energy-adjusted values of dietary component intake, to minimize the effect of writing errors. Third, we were unable to include the intake of dietary supplements in calculating intake of fatty acids and vitamin D, given that reliable data for composition of dietary supplements were not available in Japan. We included dietary supplement use (yes or no) as a confounder. Fourth, although we attempted to adjust for a wide range of potential confounding variables, we were unable to rule out residual ones. Fifth, we did not perform blood tests. Sixth, we did not assess the impact of sedentary behavior and physical activity. And finally, the study has a cross-sectional design, precluding assessment of causal effects of fatty acid or vitamin D intake on the prevalence of MGD.

## 5. Conclusions

We have performed a population-based study to assess the relationship between dietary intake of fatty acids and vitamin D and the prevalence of MGD among residents of Takushima Island, Japan. Our results suggest that high intake of total fat, SFAs, oleic acid, and vitamin D may be inversely associated with the prevalence of MGD in Japanese individuals. No significant association was detected between the prevalence of MGD and either dietary intake of n-3 PUFAs or n-6 PUFAs or the n-6/n-3 PUFA ratio by multivariate logistic regression analysis. Further epidemiological studies are warranted to clarify dietary strategies for prevention of MGD.

## Figures and Tables

**Table 1:** Characteristics of adult subjects with or without meibomian gland dysfunction (MGD) in the Hirado–Takushima study.

Characteristic	Total (*n* = 300)	Non-MGD (*n* = 194)	MGD (*n* = 106)	*p*
Age (years)	61.7 ± 15.6	57.9 ± 15.7	68.2 ± 12.8	<0.001 **
Male sex, *n* (%)	109 (36.3)	59 (30.4)	50 (47.2)	0.006 ^†^
Body height (cm)	158.2 ± 8.2	158.5 ± 8.1	157.7 ± 8.3	0.51
Body weight (kg)	60.2 ± 11.6	69.5 ± 12.2	59.5 ± 10.5	0.72
Body mass index (kg/m^2^)	23.9 ± 3.5	24.0 ± 3.5	23.8 ± 3.4	0.99
Occupation, *n* (%)				
Fisherman	38 (12.7)	27 (13.9)	11 (10.4)	0.52
Farmer	28 (9.3)	8 (4.1)	5 (4.7)	
Local government official	13 (4.3)	15 (7.7)	13 (12.3)	
Other	221 (73.7)	144 (74.2)	77 (72.6)	
History of ocular surgery, *n* (%)	51 (17.0)	27 (13.9)	24 (22.6)	0.076
Eyedrop use, *n* (%)	131 (43.7)	81 (41.8)	50 (47.2)	0.40
Contact lens wear, *n* (%)	15 (5.0)	13 (6.7)	2 (1.9)	0.095
History of chronic systemic disease, *n* (%)	171 (57.0)	93 (47.9)	78 (73.6)	<0.001 ^††^
History of dyslipidemia, *n* (%)	13 (4.3)	7 (3.6)	6 (5.7)	0.39
Taking lipid-lowering agents, *n* (%)	3 (1.0)	2 (1.0)	1 (0.9)	1.0
Current smoking, *n* (%)	26 (8.7)	19 (9.8)	7 (6.6)	0.40
Alcohol drinking, *n* (%)	254 (84.7)	165 (85.1)	89 (84.0)	0.87
Dietary supplement use, *n* (%)	77 (25.7)	49 (25.3)	28 (26.4)	0.89

Data are means ± standard deviations or *n* (%), as indicated. *p* values for comparisons between the non-MGD and MGD groups were determined with the Mann–Whitney *U* test (age, body height, body weight, and body mass index; ** *p* < 0.001) or Fisher’s exact test (other characteristics; ^†^ *p* < 0.05, ^††^ *p* < 0.001).

**Table 2:** Daily intake of dietary components in adult subjects with or without meibomian gland dysfunction (MGD) in the Hirado–Takushima study.

Dietary Component	Total (*n* = 300)	Non-MGD (*n* = 194)	MGD (*n* = 106)	*p*
Energy intake (kcal/day)	1782.9 ± 564.3	1744.6 ± 550.4	1853.0 ± 585.0	0.057
Total fat (g/day)	50.7 ± 11.7	52.1 ± 11.1	48.0 ± 12.4	0.007 *
Animal fat (g/day)	23.3 ± 8.3	24.5 ± 8.3	21.0 ± 8.0	0.002 *
Plant fat (g/day)	27.4 ± 7.2	27.6 ± 6.9	27.0 ± 7.9	0.60
Saturated fatty acids (g/day)	13.7 ± 4.0	14.2 ± 4.0	12.8 ± 3.9	0.015 *
Monounsaturated fatty acids (g/day)	18.1 ± 4.5	18.7 ± 4.2	17.1 ± 4.7	0.005 *
Oleic acid (g/day)	16.4 ± 4.1	16.9 ± 3.9	15.5 ± 4.3	0.007 *
Polyunsaturated fatty acids (g/day)	12.3 ± 2.9	12.6 ± 2.8	11.8 ± 3.1	0.039 *
n-3 Polyunsaturated fatty acids (g/day)	2.6 ± 1.0	2.7 ± 1.0	2.5 ± 0.9	0.017 *
α-Linolenic acid (g/day)	1.5 ± 0.4	1.6 ± 0.4	1.5 ± 0.5	0.10
Eicosapentaenoic acid (g/day)	0.33 ± 0.27	0.35 ± 0.30	0.30 ± 0.22	0.044 *
Docosahexaenoic acid (g/day)	0.55 ± 0.40	0.58 ± 0.44	0.50 ± 0.33	0.051
n-6 Polyunsaturated fatty acids (g/day)	9.7 ± 2.4	9.8 ± 2.3	9.3 ± 2.5	0.10
Linoleic acid (g/day)	9.4 ± 2.3	9.5 ± 2.2	9.1 ± 2.5	0.11
Arachidonic acid (g/day)	0.16 ± 0.05	0.17 ± 0.05	0.16 ± 0.06	0.17
n-6/n-3 Polyunsaturated fatty acid ratio	4.1 ± 1.3	4.0 ± 1.2	4.0 ± 1.6	0.40
Cholesterol (mg/day)	377.3 ± 132.6	381.8 ± 126.6	369.0 ± 143.1	0.49
Vitamin D (µg/day)	15.4 ± 11.9	16.4 ± 13.0	13.6 ± 9.6	0.039 *

Data are means ± standard deviations. Nutrient intake was adjusted for energy according to the residual method. *p* values for comparisons between the non-MGD and MGD groups were determined with the Mann–Whitney *U* test (* *p* < 0.05).

**Table 3:** Contribution of each food group to total fat and vitamin D estimated by a brief-type diet history questionnaire in adult subjects of the Hirado–Takushima study.

Food Group	Total Fat (%)	Vitamin D (%)
Cooking oil	20.2 ± 9.1	0.0 ± 0.0
Animal food		
Meat	15.4 ± 10.9	0.4 ± 4.7
Fish and shellfish	11.8 ± 10.1	92.4 ± 55.4
Dairy products	10.8 ± 8.7	0.7 ± 11.2
Eggs	7.4 ± 5.0	3.8 ± 34.8
Plant food		
Seasonings and spices	10.3 ± 7.6	0.3 ± 1.9
Confectionaries	8.9 ± 9.6	0.6 ± 13.7
Cereals	7.5 ± 4.3	0.2 ± 1.9
Pulses	6.7 ± 4.1	0.0 ± 0.0
Vegetables	0.8 ± 0.5	1.5 ± 5.1
Fruits	0.2 ± 0.3	0.0 ± 0.0
Potatoes	0.1 ± 0.1	0.0 ± 0.0

Data are mean ± standard deviations.

**Table 4:** Multivariate adjusted odds ratios (ORs) and 95% confidence intervals (CIs) for the prevalence of meibomian gland dysfunction (MGD) by quintile (Q) of intake of specific fats and vitamin D among adult subjects in the Hirado–Takushima study.

			Q1 (Lowest)	Q2	Q3	Q4	Q5 (Highest)	*p* for Trend
Dietary Component			*n* = 60)	(*n* = 60)	(*n* = 60)	(*n* = 60)	*n* = 60)	
Total fat								
	Intake	(g/day)	≤41.5	41.5–47.7	47.9–53.9	54.1–61.7	≥61.7	
	MGD	(%)	48.3	31.7	41.7	33.3	21.7	
	Model 1	OR (95% CI)	1.00	0.61 (0.28–1.34)	1.07 (0.49–2.34)	0.94 (0.42–2.13)	0.46 (0.20–1.07)	0.20
	Model 2	OR (95% CI)	1.00	0.60 (0.27–1.35)	1.02 (0.46–2.26)	0.86 (0.37–1.97)	0.40 (0.16–0.97)	0.16
Animal fat								
	Intake	(g/day)	≤16.9	16.9–21.0	21.2–25.0	25.1–29.8	≥29.8	
	MGD	(%)	46.7	40.0	36.7	30.0	23.3	
	Model 1	OR (95% CI)	1.00	0.92 (0.42–2.00)	0.76 (0.35–1.66)	0.87 (0.38–1.97)	0.59 (0.25–1.36)	0.75
	Model 2	OR (95% CI)	1.00	0.93 (0.42–2.06)	0.69 (0.31–1.53)	0.75 (0.32–1.76)	0.55 (0.23–1.32)	0.66
Plant fat								
	Intake	(g/day)	≤21.5	21.9–25.8	25.9–28.7	28.9–33.0	≥33.1	
	MGD	(%)	41.7	35.0	36.7	23.3	40.0	
	Model 1	OR (95% CI)	1.00	0.87 (0.39–1.93)	1.10 (0.49–2.46)	0.64 (0.27–1.49)	1.09 (0.50–2.39)	0.70
	Model 2	OR (95% CI)	1.00	0.79 (0.35–1.79)	0.99 (0.43–2.28)	0.54 (0.22–1.30)	0.95 (0.42–2.16)	0.58
Saturated fatty acids								
	Intake	(g/day)	≤10.2	10.2–12.6	12.6–14.5	14.5–16.7	≥16.8	
	MGD	(%)	53.3	26.7	31.7	41.7	23.3	
	Model 1	OR (95% CI)	1.00	0.34 (0.15–0.77)	0.55 (0.25–1.21)	1.04 (0.47–2.28)	0.46 (0.20–1.09)	0.026 *
	Model 2	OR (95% CI)	1.00	0.34 (0.15–0.78)	0.44 (0.19–1.02)	0.94 (0.42–2.13)	0.40 (0.17–0.97)	0.020 *
Monounsaturated fatty acids								
	Intake	(g/day)	≤14.4	14.5–17.1	17.1–19.5	19.5–22.1	≥22.1	
	MGD	(%)	46.7	30.0	50.0	23.3	26.7	
	Model 1	OR (95% CI)	1.00	0.57 (0.25–1.27)	1.33 (0.62–2.88)	0.62 (0.27–1.46)	0.58 (0.25–1.30)	0.13
	Model 2	OR (95% CI)	1.00	0.55 (0.24–1.25)	1.27 (0.58–2.80)	0.58 (0.24–1.38)	0.50 (0.21–1.17)	0.094
Oleic acid								
	Intake	(g/day)	≤13.3	13.4–15.2	15.3–17.6	17.6–19.9	≥19.9	
	MGD	(%)	48.3	30.0	46.7	28.3	23.3	
	Model 1	OR (95% CI)	1.00	0.50 (0.22–1.11)	1.14 (0.53–2.49)	0.74 (0.33–1.69)	0.46 (0.20–1.07)	0.10
	Model 2	OR (95% CI)	1.00	0.54 (0.24–1.21)	1.08 (0.49–2.39)	0.68 (0.29–1.58)	0.40 (0.17–0.97)	0.11
Polyunsaturated fatty acids								
	Intake	(g/day)	≤9.9	10.0–11.5	11.5–13.1	13.2–14.7	≥14.7	
	MGD	(%)	45.0	35.0	40.0	31.7	25.0	
	Model 1	OR (95% CI)	1.00	0.82 (0.37–1.82)	1.20 (0.54–2.64)	0.93 (0.41–2.12)	0.51 (0.22–1.15)	0.31
	Model 2	OR (95% CI)	1.00	0.79 (0.35–1.78)	1.16 (0.51–2.62)	0.83 (0.36–1.93)	0.46 (0.19–1.07)	0.25
n-3 Polyunsaturated fatty acids								
	Intake	(g/day)	≤1.9	1.9–2.4	2.4–2.7	2.7–3.1	≥3.2	
	MGD	(%)	45.0	48.3	23.3	31.7	28.3	
	Model 1	OR (95% CI)	1.00	1.33 (0.60–2.94)	0.45 (0.19–1.05)	0.66 (0.29–1.48)	0.53 (0.24–1.18)	0.049 *
	Model 2	OR (95% CI)	1.00	1.29 (0.57–2.89)	0.47 (0.20–1.12)	0.64 (0.28–1.47)	0.52 (0.23–1.18)	0.077
α-Linolenic acid								
	Intake	(g/day)	≤1.2	1.2–1.4	1.4–1.6	1.6–1.9	≥1.9	
	MGD	(%)	45.0	33.3	36.7	31.7	30.0	
	Model 1	OR (95% CI)	1.00	0.71 (0.32–1.57)	1.01 (0.45–2.23)	0.75 (0.34–1.68)	0.67 (0.30–1.50)	0.77
	Model 2	OR (95% CI)	1.00	0.75 (0.34–1.67)	0.96 (0.42–2.18)	0.68 (0.30–1.55)	0.62 (0.27–1.43)	0.74
Eicosapentaenoic acid								
	Intake	(g/day)	≤0.15	0.15–0.26	0.26–0.34	0.34–0.47	≥0.47	
	MGD	(%)	45.0	40.0	35.0	21.7	35.0	
	Model 1	OR (95% CI)	1.00	0.87 (0.39–1.91)	0.90 (0.40–2.02)	0.40 (0.17–0.94)	0.63 (0.29–1.36)	0.20
	Model 2	OR (95% CI)	1.00	0.84 (0.37–1.87)	0.93 (0.41–2.12)	0.41 (0.17–0.97)	0.68 (0.31–1.50)	0.24
Docosahexaenoic acid								
	Intake	(g/day)	≤0.28	0.28–0.44	0.44–0.57	0.57–0.75	≥0.76	
	MGD	(%)	43.3	46.7	23.3	31.7	31.7	
	Model 1	OR (95% CI)	1.00	1.18 (0.54–2.58)	0.55 (0.24–1.30)	0.70 (0.31–1.56)	0.57 (0.26–1.26)	0.24
	Model 2	OR (95% CI)	1.00	1.20 (0.54–2.66)	0.58 (0.24–1.38)	0.74 (0.33–1.67)	0.61 (0.28–1.37)	0.34
n-6 Polyunsaturated fatty acids								
	Intake	(g/day)	≤7.7	7.7–9.2	9.2–10.2	10.2–11.5	≥11.5	
	MGD	(%)	43.3	33.3	40.0	31.7	28.3	
	Model 1	OR (95% CI)	1.00	0.79 (0.36–1.76)	1.22 (0.55–2.71)	1.54 (0.70–3.41)	0.97 (0.43–2.02)	0.74
	Model 2	OR (95% CI)	1.00	0.71 (0.31–1.62)	1.16 (0.51–2.63)	0.82 (0.35–1.92)	0.63 (0.27–1.49)	0.61
Linoleic acid								
	Intake	(g/day)	≤7.4	7.5–8.9	8.9–9.9	9.9–11.1	≥11.2	
	MGD	(%)	43.3	31.7	41.7	31.7	28.3	
	Model 1	OR (95% CI)	1.00	0.72 (0.32–1.62)	1.31 (0.59–2.90)	1.00 (0.44–2.28)	0.74 (0.32–1.68)	0.57
	Model 2	OR (95% CI)	1.00	0.66 (0.29–1.50)	1.25 (0.56–2.83)	0.83 (0.35–1.94)	0.63 (0.27–1.49)	0.44
Arachidonic acid								
	Intake	(g/day)	≤0.12	0.12–0.15	0.15–0.17	0.17–0.20	≥0.20	
	MGD	(%)	45.0	38.3	21.7	36.7	35.0	
	Model 1	OR (95% CI)	1.00	0.87 (0.40–1.91)	0.42 (0.18–0.99)	0.95 (0.43–2.11)	0.78 (0.35–1.69)	0.27
	Model 2	OR (95% CI)	1.00	0.94 (0.42–2.09)	0.44 (0.18–1.03)	0.46 (0.20–1.10)	1.00 (0.44–2.25)	0.26
n-6/n-3 Polyunsaturated fatty acid ratio								
	Ratio		≤3.2	3.2–3.6	3.6–4.0	4.0–4.7	≥4.7	
	MGD	(%)	36.7	31.7	28.3	38.3	41.7	
	Model 1	OR (95% CI)	1.00	0.89 (0.40–1.99)	0.97 (0.43–2.21)	1.44 (0.65–3.21)	1.53 (0.70–3.34)	0.58
	Model 2	OR (95% CI)	1.00	0.86 (0.38–1.95)	0.93 (0.40–2.17)	1.36 (0.60–3.07)	1.32 (0.59–2.95)	0.75
Cholesterol								
	Intake	(mg/day)	≤270.6	270.8–330.7	331.2–395.0	399.1–479.8	≥480.6	
	MGD	(%)	40.0	35.0	26.7	40.0	35.0	
	Model 1	OR (95% CI)	1.00	1.04 (0.47–2.33)	0.63 (0.27–1.45)	1.41 (0.63–3.14)	0.73 (0.33–1.63)	0.33
	Model 2	OR (95% CI)	1.00	1.07 (0.47–2.44)	0.60 (0.25–1.41)	1.36 (0.60–3.11)	0.70 (0.31–1.58)	0.30
Vitamin D								
	Intake	(μg/day)	≤7.6	7.6–11.7	11.7–14.9	15.0–22.3	≥22.6	
	MGD	(%)	46.7	35.0	38.3	28.3	28.3	
	Model 1	OR (95% CI)	1.00	0.55 (0.24–1.23)	0.98 (0.44–2.20)	0.55 (0.24–1.24)	0.38 (0.17–0.86)	0.087
	Model 2	OR (95% CI)	1.00	0.56 (0.25–1.27)	1.00 (0.44–2.27)	0.51 (0.22–1.19)	0.38 (0.17–0.87)	0.081

Nutrient intake was adjusted for energy by the residual method. Model 1: adjusted for age (years, continuous) and sex (male or female). Model 2: adjusted for age (years, continuous), sex (male or female), body mass index (kg/m^2^, continuous), history of chronic systemic disease (yes or no), and alcohol drinking (yes or no). * *p* < 0.05.

## Data Availability

The data presented in this study are available on request from the corresponding author.
